# Trajectory of COVID-19 Vaccine Hesitancy Over Time and Association of Initial Vaccine Hesitancy With Subsequent Vaccination

**DOI:** 10.1001/jamanetworkopen.2021.26882

**Published:** 2021-09-24

**Authors:** Aaron J. Siegler, Nicole Luisi, Eric W. Hall, Heather Bradley, Travis Sanchez, Benjamin A. Lopman, Patrick S. Sullivan

**Affiliations:** 1 Department of Epidemiology, Rollins School of Public Health, Emory University, Atlanta, Georgia; 2 Department of Epidemiology, Georgia State University, Atlanta

## Abstract

This cohort study assesses the association between baseline vaccine hesitancy and vaccine receipt at study follow-up and explores the validity of vaccine self-report.

## Introduction

Vaccine hesitancy is a critical barrier to achieving high COVID-19 vaccine coverage.^1,2^ The stability of hesitancy over time is unclear, as is the association between hesitancy and eventual vaccine receipt. Moreover, despite widespread use, the validity of self-reported COVID-19 vaccine receipt has not been established. Using a population-based, serosurvey cohort in the US, we assessed the association between baseline vaccine hesitancy and vaccine receipt at study follow-up and explored the validity of vaccine self-report.

## Methods

This cohort study, combined with study methods published elsewhere,^3^ follows the Strengthening the Reporting of Observational Studies in Epidemiology (STROBE) reporting guideline. The study was approved by the Emory University institutional review board. All study participants completed an online informed consent procedure.

As described elsewhere,^3^ participants were recruited from a national address-based frame. At baseline (August 9 to December 8, 2020) and follow-up (March 2 to April 21, 2021), surveys measured COVID-19 vaccine hesitancy, and biological specimens measured antibody response. Validation analysis compared anti-spike IgG (Platelia Total Antibody Assay; Bio-Rad) with self-reported vaccination status. Vaccine self-report was assessed with the question, “Have you received a COVID-19 vaccine?” with responses of “Yes, one dose,” “Yes, two doses,” and “No.” Vaccine hesitancy was assessed with responses of “very unlikely,” “unlikely,” or “unsure” categorized as hesitant and “likely” or “very likely” categorized as willing. Sociodemographic variables, including race and ethnicity, were self-reported and were collected as part of the cohort study. Design weights were adjusted using classification and regression tree analysis and a raking procedure. Weighted estimates and 2-sided 95% CIs were developed in SAS statistical software version 9.4 (SAS Institute), with alluvial plots conducted in R statistical software version 4.0.5 (R Project for Statistical Computing).

## Results

Of 4654 baseline respondents (2727 women [59%]; mean [SD] age, 50.7 [17.2] years), a total of 3439 (74%) completed follow-up (Table). Alluvial plots show the path of persons from baseline hesitancy status to follow-up vaccination status, with the widths corresponding to proportions observed (Figure). Among persons hesitant to vaccinate at baseline, at follow-up, 32% (95% CI, 27%-37%) reported receiving 1 or more vaccine doses, 37% (95% CI, 32%-42%) reported being likely to be vaccinated, and 32% (95% CI, 27%-37%) remained unlikely to be vaccinated. In contrast, among persons likely to be vaccinated at follow-up, 54% (95% CI, 50%-57%) had received 1 or more vaccine doses, 39% (95% CI, 36%-43%) remained likely to be vaccinated, and 7% (95% CI, 5%-9%) reported being unlikely to be vaccinated. Baseline vaccine willingness was higher among persons with a bachelor’s or graduate degree than among persons with lower education (76% [95% CI, 72%-78%] vs 65% [95% CI, 61%-69%]), and at follow-up these differences were reflected in vaccination, with vaccination rates of 54% (95% CI, 51%-58%) vs 43% (95% CI, 39%-47%). For Hispanic individuals, baseline vaccine willingness was similar to that of non-Hispanic White individuals (71% [95% CI, 64%-78%] vs 69% [95% CI, 66%-72%]), yet at follow-up fewer Hispanic individuals than non-Hispanic White individuals were vaccinated (31% [95% CI, 25%-38%] vs 51% [95% CI, 47%-54%]). Because not all participants were eligible for vaccines at follow-up, we conducted a sensitivity analysis restricted to participants reporting vaccine eligibility and found that among persons hesitant to vaccinate at baseline, at follow-up 51% reported receiving 1 or more vaccine dose, 22% reported being likely to be vaccinated, and 27% remained unlikely to be vaccinated.

**Table.** Current COVID-19 Vaccination Status and Likelihood of Future Vaccination by Baseline COVID-19 Vaccine Hesitancy Among Participants in a Population-Based Longitudinal US Cohort, 2020-2021^a^

Variable	Participants, % (95% CI) (N = 3439)^b^					
	Baseline hesitant to be vaccinated (n = 1061)			Baseline likely to be vaccinated (n = 2378)		
	Vaccinated at follow-up (n = 389)	Follow-up likely to be vaccinated (n = 384)	Follow-up unlikely to be vaccinated (n = 288)^c^	Vaccinated at follow-up (n = 1395)	Likely to be vaccinated at follow-up (n = 859)	Unlikely to be vaccinated at follow-up (n = 124)^c^
Overall	32 (27-37)	37 (32-42)	32 (27-37)	54 (50-57)	39 (36-43)	7 (5-9)
Sex						
Male	27 (20-35)	44 (36-53)	29 (21-38)	49 (44-54)	45 (40-50)	6 (3-9)
Female	35 (30-41)	31 (26-37)	33 (28-40)	58 (53-63)	34 (29-38)	8 (6-11)
Race/ethnicity						
Hispanic	18 (11-28)	50 (37-62)	33 (21-47)	36 (28-45)	54 (45-63)	10 (5-18)
Non-Hispanic Asian	65 (44-81)	22 (10-44)	13 (5-29)	42 (30-54)	53 (40-65)	6 (2-18)
Non-Hispanic Black	34 (23-48)	39 (26-53)	27 (16-41)	56 (43-69)	43 (31-56)	1 (0-4)
Non-Hispanic other^d^	43 (20-70)	24 (9-50)	33 (12-64)	78 (61-89)	15 (7-30)	7 (2-23)
Non-Hispanic White	32 (27-38)	34 (29-41)	33 (28-40)	59 (55-63)	34 (30-38)	7 (5-10)
Age, y						
18-34	12 (7-20)	47 (37-58)	41 (31-51)	34 (27-41)	57 (50-64)	9 (6-15)
35-44	25 (17-36)	41 (30-52)	34 (24-46)	39 (32-47)	51 (43-59)	10 (5-17)
45-54	18 (11-28)	41 (31-53)	41 (30-52)	44 (35-52)	47 (39-56)	9 (5-17)
55-64	48 (37-60)	29 (21-40)	23 (15-33)	53 (45-61)	43 (36-51)	4 (2-8)
≥65	73 (60-83)	15 (8-27)	12 (6-23)	91 (86-94)	6 (4-10)	3 (1-6)
Education						
High school, general equivalency diploma, or less	24 (16-33)	39 (29-49)	38 (28-48)	52 (45-60)	37 (30-45)	11 (7-16)
Some college or associate’s degree	31 (24-38)	35 (29-43)	34 (27-42)	49 (43-56)	42 (36-48)	9 (6-13)
Bachelor’s degree	41 (33-50)	37 (29-45)	22 (16-30)	51 (46-57)	46 (40-52)	3 (1-7)
Graduate degree	50 (38-62)	32 (21-45)	18 (11-28)	68 (63-73)	30 (25-35)	2 (1-4)
Annual household income, $						
0-24 999	22 (14-32)	47 (36-59)	31 (21-44)	36 (27-47)	54 (43-64)	10 (5-19)
25 000-49 999	28 (19-39)	36 (26-48)	36 (26-48)	52 (44-61)	34 (26-43)	13 (8-22)
50 000-99 999	31 (23-39)	37 (29-47)	32 (24-42)	57 (50-64)	38 (32-45)	5 (3-8)
100 000-199 999	37 (28-47)	34 (26-44)	29 (20-39)	55 (48-61)	39 (33-45)	6 (4-11)
≥200 000	49 (33-65)	23 (12-38)	29 (15-48)	57 (49-66)	38 (30-47)	4 (1-12)
Urbanicity						
Metropolitan	31 (26-36)	31 (26-37)	38 (33-44)	52 (49-56)	40 (37-44)	7 (5-10)
Micropolitan or rural	38 (26-52)	33 (22-46)	29 (18-43)	65 (54-74)	32 (22-43)	4 (2-8)
Household size						
1-2 persons	40 (34-46)	33 (27-39)	28 (22-34)	63 (58-67)	32 (28-36)	5 (3-7)
≥3 persons	22 (16-29)	42 (34-50)	37 (29-45)	41 (36-47)	49 (44-55)	9 (6-14)

^a^ Baseline data were collected from August 9 to December 8, 2020, and follow-up data were collected from March 2 to April 21, 2021.

^b^ Percentages and 95% CIs are weighted to the US population.

^c^ Unlikely to be vaccinated refers to persons rating themselves as either unlikely or unsure whether they would be vaccinated.

^d^ Non-Hispanic other refers to non-Hispanic persons identifying as American Indian or Alaska Native, Native Hawaiian or other Pacific Islander, any other race, or prefer not to answer.

**Figure.**
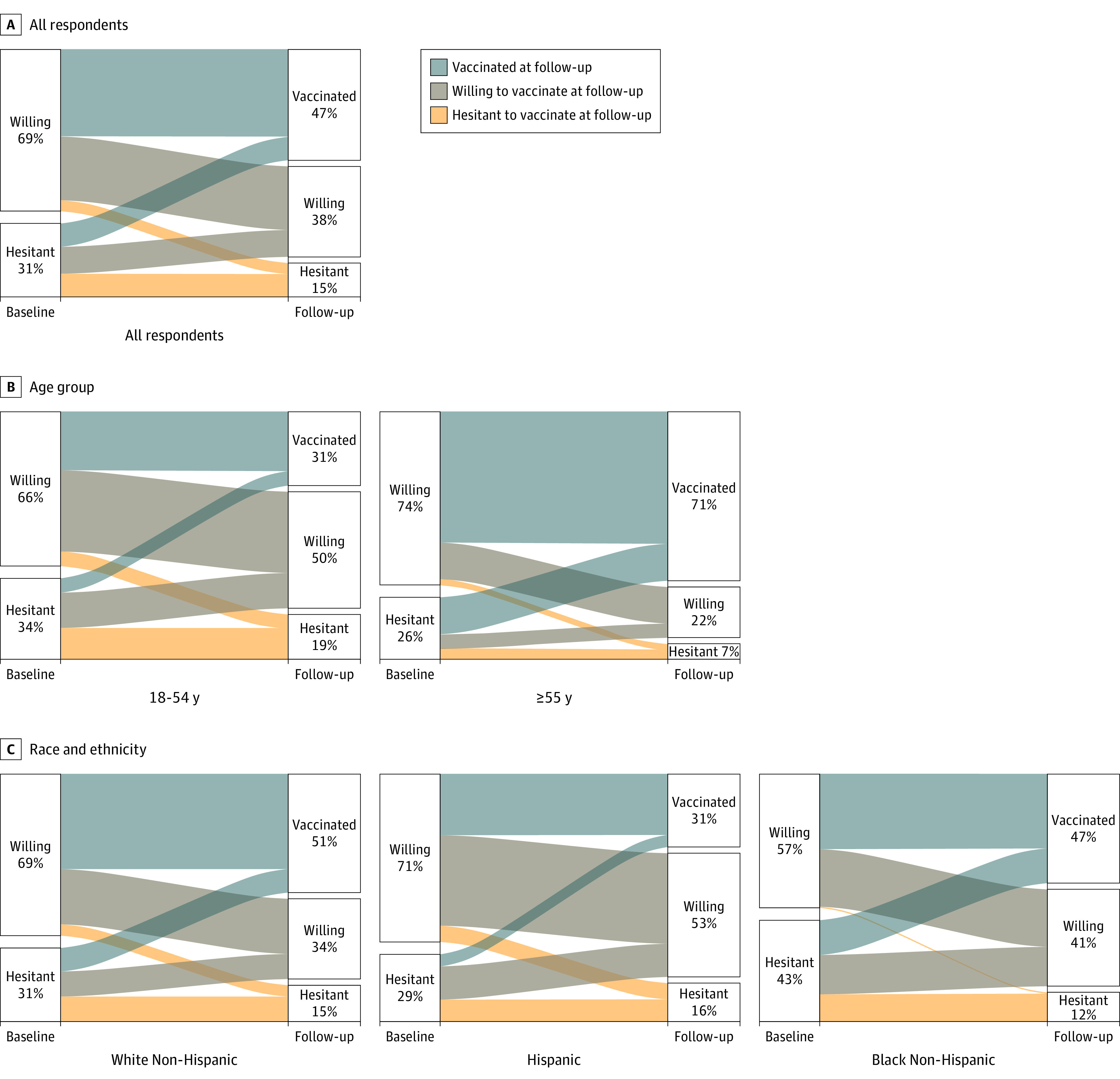
Alluvial Plot Paths From Baseline Hesitancy to Follow-up Vaccination Status Among a National, Weighted Sample of 3439 US Respondents, 2020-2021 The baseline was from August 9 to December 8, 2020, and follow-up was from March 2 to April 21, 2021. Data are shown for all respondents (A), by age group (B), and by race and ethnicity (C). The full breakdown of categories is provided in the Table.

Self-reported vaccine receipt indicated substantial validity compared with the reference standard detection of anti-spike IgG among 1949 participants (excluding 378 with natural infection and 868 without full dosing or with unknown vaccine manufacturer). Self-report had 98.2% (638 of 650 respondents) positive predictive value, 97.3% (35 of 1299 respondents) negative predictive value, 94.8% (638 of 673 respondents) sensitivity, and 99.1% (12 of 1276 respondents) specificity.

## Discussion

This cohort study found that COVID-19 vaccine hesitancy is not a stable trait precluding vaccination but, instead, is labile. Hesitancy decreased between late 2020 and early 2021, with nearly one-third (32%) of persons who were initially hesitant being vaccinated at follow-up and more than one-third (37%) transitioning from vaccine hesitant into vaccine willing. Early plans regarding vaccination frequently deviated from later action in vaccine seeking. Self-reported vaccination status was congruent with biological tests, indicating that it is a valid metric. Changes in hesitancy have not alleviated health inequities in vaccines received, and further studies are needed to explore the reasons why vaccine hesitancy is changing over time by group. Our analysis is limited in that vaccines were not available to all respondents until April 19, 2021, and our follow-up period ended before this date.

## Conclusions

Vaccine hesitancy is waning, yet inequities in receipt remain. There is a clear public health opportunity to convert higher vaccine willingness into successfully delivered vaccinations.
